# Analysis of ultrasound features and clinical manifestations of struma ovarii

**DOI:** 10.3389/fonc.2026.1721601

**Published:** 2026-03-19

**Authors:** Liang Hou, Yixin Li, Feixue Zhang, Lulu Zhang, Xiuliang Wei, Mei Wu

**Affiliations:** 1 Department of Ultrasound, The Second Qilu Hospital of Shandong University, Jinan, Shandong, China; 2 Department of Medical Imaging, The Second Qilu Hospital of Shandong University, Jinan, Shandong, China; 3 Department of Pathology, The Second Qilu Hospital of Shandong University, Jinan, Shandong, China

**Keywords:** clinical manifestation, analysis, struma ovarii, ultrasonography, ultrasound features

## Abstract

**Objective:**

To analyze the clinical manifestations and ultrasonographic features of struma ovarii.

**Methods:**

The clinical data, ultrasound characteristics and serological tumor markers of 20 cases of struma ovarii, confirmed by postoperative pathological examination at our hospital, were retrospectively analyzed.

**Results:**

A total of 20 patients with benign struma ovarii were enrolled for the present study. None of the patients presented with ascites, and tumor markers were within normal ranges in all cases. Among the included patients,18 were over 30 years of age, 18 had cystic or cystic-solid masses, 19 had regular morphology, and 18 had well-defined borders without adhesion to surrounding tissues. Of note, 11 patients had irregular band-like septation within the masses, nine showed mural nodules, and six had “thyroid pearls” inside the masses. All patients underwent laparoscopic surgery, and no postoperative recurrences were observed.

**Conclusion:**

The clinical manifestations of struma ovarii are nonspecific, and its ultrasonographic features are heterogeneous. Most masses are cystic or cystic-solid, with regular morphology, well-defined borders, and no adhesion to surrounding tissues, with some containing internal septa or solid mural nodules. Among the observed features, the presence of “thyroid pearls” represents a relatively specific finding.

## 1 Introduction

Struma ovarii (SO) is a rare and distinct type of mature ovarian teratoma, accounting for approximately 0.1% of all ovarian tumors and 1%–3% of all ovarian teratomas (1–3), with less than 0.5% being malignant (4, 5). Ultrasound, a fundamental imaging modality, plays a crucial role in evaluating gynecological tumors. Nevertheless, due to the rarity of SO, few cases have been reported, and their clinical and ultrasonographic features remain insufficiently characterized. Furthermore, the ultrasound findings of SO lack specificity, resulting in a low diagnostic accuracy, and most cases cannot be definitively diagnosed preoperatively (6).Therefore, systematically summarizing the clinical manifestations and ultrasound features of SO, along with identifying imaging characteristics of differential diagnostic value, is essential for optimizing preoperative diagnostic strategies. In the present study, we retrospectively analyzed the clinical data of 20 patients with SO treated at the Qilu Second Hospital of Shandong University, summarized their clinical and ultrasonographic features, and aimed to improve the understanding of this condition through ultrasound. Moreover, we aim to provide a basis for early clinical identification, accurate staging, and individualized diagnostic and treatment planning, thereby improving patient prognosis.

## 2 Materials and methods

### 2.1 Patients

A total of 20 cases were included, all of whom underwent ultrasound examination in the Second Qilu Hospital of Shandong University between December 2015 and June 2023 and were confirmed to have SO by postoperative pathological diagnosis. The inclusion criteria were as follows (1): Complete preoperative of the uterus and bilateral ovaries ultrasound examination at Second Qilu Hospital of Shandong University, (2) pathologically verified SO, and (3) complete clinical information and ultrasound images. The exclusion criteria included the following: (1) Incomplete clinical information or ultrasound images, (2) patients who had undergone other gynecological surgeries before the examination, and (3) pathological results indicating SO with other malignant masses. Clinical information was obtained from the hospital’s medical record system and included age, medical history, family history, reason for presentation, age at menarche, menopausal status, number of pregnancies, and tumor markers (including CA125, CA19-9, AFP, and CEA).

### 2.2 Examination methods

Ultrasonography was performed using an abdominal probe (GE Voluson E8, GE) with a frequency of 5 MHz and a transvaginal probe (GE Voluson E8, GE) with a frequency of 8 MHz. All patients underwent transabdominal and/or transvaginal ultrasound examinations. The characteristics of ovarian masses were evaluated from multiple planes and angles, and relevant measurements and images were recorded. Observations included the location, size, echogenicity (Hyperechoic and hypoechoic areas are defined as regions that appear brighter or darker, respectively, than the normal ovarian parenchyma on ultrasound.), posterior acoustic shadowing, presence of septations and protrusions, shape, border definition, relationship with surrounding structures, presence of calcifications, ascites and Color Doppler Flow Imaging (CDFI). The Adler semi-quantitative method CDFI was used to classify the blood flow signal of the nodule into four levels (7): Grade 0: No blood flow within the nodule; grade I: Minimal blood flow, with 1–2 rod-shaped and punctate blood flow within the nodule; grade II: Moderate blood flow, with 1 long or 3–4 punctate blood vessels within the nodule; and grade III: Rich blood flow, with 2 long or more than 5 punctate blood vessels within the nodule. All images were independently reviewed by two ultrasound physicians with over five years of experience. In cases of disagreement, consensus was reached through consultation with a higher-level physician, and the findings were documented.

### 2.3 Statistical analysis

Data analysis was performed using SPSS 26.0 software. Continuous variables with a normal distribution were expressed as mean ± standard deviation (x̄ ± s); non-normally distributed variables were presented as median.

## 3 Results

### 3.1 Clinical features and surgical procedures

Among the 20 patients included in the analysis, all had solitary masses, totaling 20 masses. Their ages ranged from 21 to 71 years, with a median age of 49.0 years. Eleven patients were asymptomatic, four reported pain, two experienced nausea, and three had palpable masses. None had a family history of the condition. Ten patients were premenopausal, and ten were postmenopausal. The age at menarche ranged from 12 to 16 years, with a median of 15.0 years. Two patients had no history of pregnancy, three had one pregnancy, and the remaining 15 had two or more pregnancies. Tumor markers, including CA125, CA19-9, AFP, and CEA, were all within normal limits. Ten patients underwent total excision of the ovarian mass, while the other ten had mass excision only, as detailed in **Table 1**.

**Table 1:** Clinical characteristics and surgical interventions.

Clinical characteristics	Struma ovarii
Age (years)	49.0 (21-71)
<30 [n (%)]	2 (10)
30-50 [n (%)]	10 (50)
>50[n (%)]	8 (40)
Medical history (months)	2 (0.6-24)
Symptoms	
No [n (%)]	11 (55)
Pain n (%)]	4 (20)
Nausea [n (%)]	2 (10)
Pelvic mass [n (%)]	3 (15)
Family history	
Yes [n (%)]	0
No [n (%)]	20 (100)
Menopause	
Yes [n (%)]	10 (50)
No [n (%)]	10 (50)
Age at menarche (years)	15 (12-16)
Pregnancy history	
None	2
1	3
≥2	15
CA125 (IU/L)	5.75-30.58 (0-35)
CA19-9 (IU/L)	3.29-38.0 (0-39)
CEA (IU/L) AFP (IU/L)	0.30-1.88 (0-10) 0.61-5.84 (0-20)
Surgery	
Laparoscopic mass excision [n (%)]	10 (50)
Laparoscopic ovariectomy combined with lumpectomy [n (%)]	10 (50)

Values ​​are median (range) or n (%).

### 3.2 Ultrasonic characteristics

**Figure 1:**
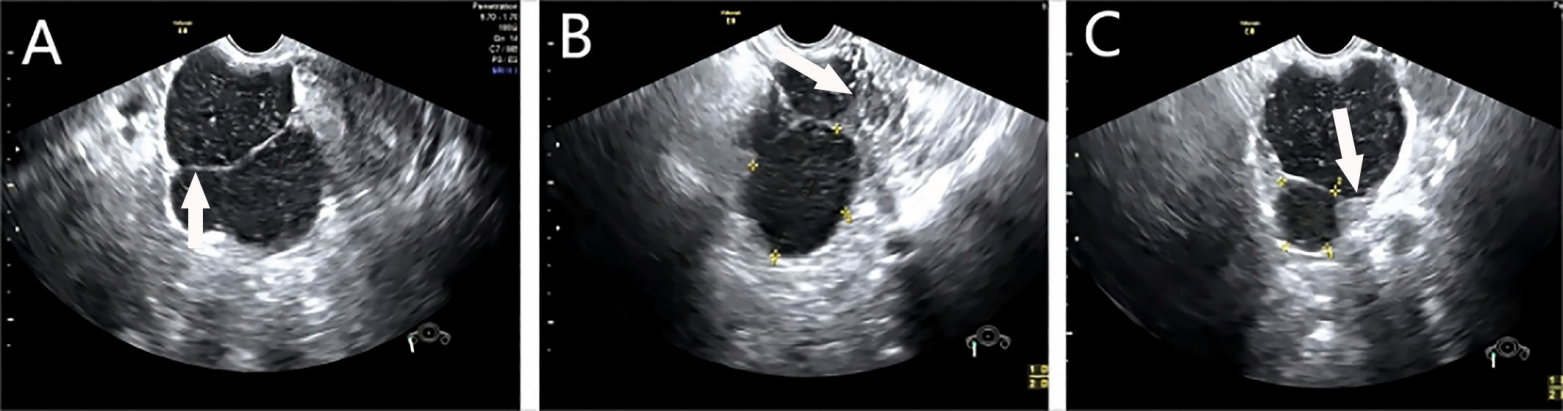
A right ovarian multilocular cystic mass measuring 13.6 x 11.8 x 10.2 cm, with multiple septations of variable thickness, focal thickening is observed in the cyst wall, poor sound transmission, and internal punctate echoes. **(A)** shows a transvaginal ultrasound image with a white arrow pointing to a septation within a large ovarian cyst. **(B)** displays another section of the cyst with an arrow indicating mural nodularity. **(C)** highlights a different view of the same cyst, with an arrow pointing to a thickened cyst wall.

**Figure 2:**
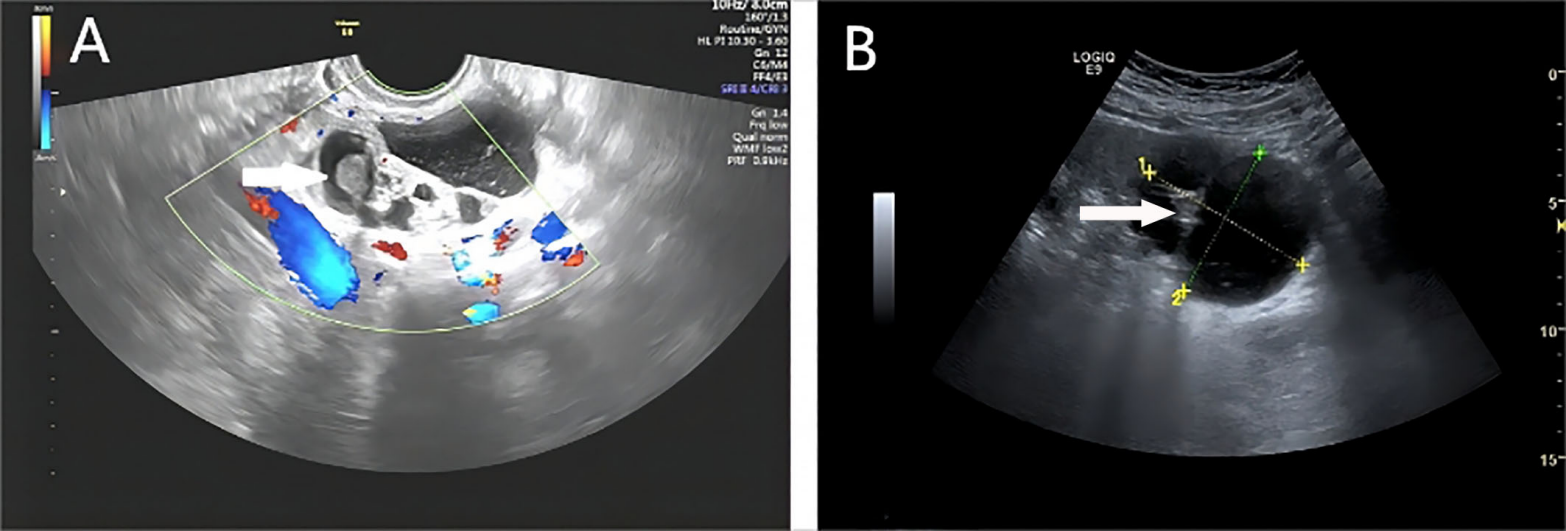
**(A)** A 62 years old patient presenting with a multilocular cystic mass in the right ovary, measuring around 6.7×6.3×4.5 cm, with poorly transmissible, fine dotted echoes and a medium-strong echogenic protrusion of about 3.5×3.0×2.5 cm in size, resembling thyroid tissue pattern, which we termed “thyroid pearl”. **(B)** A 21 years old patient with a mass on the left ovary exhibiting bicompartmental cystic echogenicity, measuring 7.0×6.1×6.1cm in size. The mass exhibited a band-like septation, within which equal echogenicity accompanied by punctate strong echogenicity was seen accompanied by “comet tail” sign, similar to thyroid tissue pattern.

All 20 masses were located in the adnexal region, with 10 cases on the left side and 10 on the right. The masses ranged in longest diameter from 2.6 to 15.3 cm, with a mean size of 7.20 ± 3.84 cm. The majority (18 of 20) were unilocular cystic, multilocular cystic, or solid cystic masses. A total of eleven masses exhibited uneven septations (**Figure 1**), seven had regular wall protrusions, and two showed irregular protrusions. Calcifications were observed in five masses. Of note, 19 masses had regular morphology; however, two masses had unclear borders, and two were adherent to surrounding tissues. Six masses contained “thyroid pearls” internally (**Figure 2**), and five masses showed blood flow signals. Ten patients underwent laparoscopic mass excision, while the other ten underwent laparoscopic ovariectomy combined with lumpectomy, as detailed in **Table 2**.

**Table 2:** Ultrasound characteristics of struma ovarii.

Ultrasound characteristics	Struma ovarii
Length(cm)	7.20 ± 3.84(2.6-15.3)
Location	
Left ovarian [n (%)]	10(50)
Right ovarian [n (%)]	10(50)
Internal echo	
Unilocular cystic [n (%)]	7 (35)
Multilocular cystic [n (%)]	3 (15)
Cystic-solid [n (%)]	8 (40)
Hyperechoic [n (%)]	1 (5)
Hypoechoic [n (%)]	1 (5)
Shadow	
With [n (%)]	4(20)
Without [n (%)]	16(80)
Cyst wall protrusion	
Without [n (%)]	11 (55)
Regular protrusion [n (%)]	7(35)
Irregular protrusion [n (%)]	2(10)
Band-like septation	
With [n (%)]	11(55)
Without [n (%)]	9(45)
Calcification	
With [n (%)]	5(25)
Without [n (%)]	15(75)
Shape	
Regular [n (%)]	19(95)
Irregular [n (%)]	1(5)
Boundary	
Distinct [n (%)]	18(90)
Unclear [n (%)]	2(10)
Relationship with surrounding areas	
Adhesion [n (%)]	2(10)
No adhesion [n (%)]	18(90)
Thyroid pearl	
With [n (%)]	6(30)
Without [n (%)]	14(70)
CDFI	
No [n (%)]	15(75)
Yes [n (%)]	5(25)

Values ​​are median (range) or n (%).

### 3.3 Ultrasound diagnosis result

In our examinations, ultrasound suggested teratoma in three cases, adenoma in two cases, granulosa cell tumor in two cases, endometriotic cyst in one case, simple cyst in six cases, and showed no specific findings in six cases.

## 4 Discussion

SO is a highly specific monodermal teratoma characterized by one of the following features (8, 9): (1) thyroid tissue comprising more than 50% of the teratoma; (2) thyroid tissue comprising less than 50% of the teratoma but with significant thyroid hormone production; or (3) grossly visible thyroid tissue within the teratoma. Although thyroid tissue is present, signs of hyperthyroidism are rare. Herein, none of the patients exhibited hyperthyroidism, consistent with previous reports (10, 11). Among our cases, only three were suspected to be teratomas on ultrasound, while six showed no specific diagnosis. The remaining cases were suspected to be adenoma, granulosa cell tumor, endometriotic cyst, or simple cyst, with no cases diagnosed as SO preoperatively. The concordance rate of preoperative ultrasound diagnosis was therefore extremely low.

Previous studies have suggested that SO typically occurs in peri-menopausal women, with ages ranging from 30 to 50 years (12). In the present study, however, the age of onset varied widely, ranging from 21 to 71 years, with a median age of 49 years. Ten patients were between 30 and 50 years old, while eight were over 50 years old. Ten patients were postmenopausal, consistent with previous findings (10, 12). We found no significant correlations between SO and family history, age at menarche, or parity, though further research is needed to explore these associations.

Up to 40% of patients with SO are asymptomatic and are incidentally diagnosed during routine examinations. The most common clinical symptoms reported are abdominal pain and distension (13–15). Here, 11 cases (55%) were incidentally discovered during physical examinations without symptoms. Four cases (20%) presented with pain, two with nausea, and three with palpable masses. Approximately one-third of patients in previous studies had ascites, while elevated CA125 levels were rare (10, 16). CA125 cannot reliably distinguish between benign and malignant adnexal masses, and its elevation may be related to the presence of ascites (17, 18). Nonetheless, none of our cases exhibited ascites, and tumor markers, including CA125, CA19-9, AFP, and CEA, were within normal ranges, in line with previous findings.

Some studies have suggested that SO occurs more frequently on the left side (19). However, in our study, all cases were solitary masses evenly distributed between the left and right adnexal areas, with 10 cases on each side, indicating no significant lateral preference. This discrepancy may be due to the limited sample size in our study. Additionally, five cases had mass diameters greater than 10 cm, with sizes ranging from 2.6 to 15.3 cm and a mean diameter of 7.20 ± 3.84 cm. These findings are consistent with previous reports, which note that SO masses are generally smaller than 10 cm in diameter (12, 20). Furthermore, our analysis showed that the solid components were small, enlarging as the cystic portion increased, this phenomenon is possibly related to the secretion of jelly-like material and cystic degeneration of the thyroid follicular epithelium within the masses.

Most masses (18/20) were cystic or cystic-solid masses, including 7 unilocular cysts, 3 multilocular cystic masses, and 8 cystic-solid masses. Both the multilocular cystic and cystic-solid types exhibited uneven septations, which may help distinguish them from adenomas, as the septations in adenomas are typically thin and uniform (18, 21). Nine cystic masses had solid protrusions, and six of these presented with hyperechoic solid components resembling “thyroid pearls,” a feature first described by Savelli et al. in 2006 (22, 23). “Thyroid pearls” were described as “ round white ball”, characterized by a smooth surface and adherence to the inner cyst wall. Pathological correlation confirmed the presence of thyroid tissue within these nodules, may represent proliferative thyroid tissue rich in blood vessels, although their clinical significance remains uncertain (24, 25). In the present study, among the identifiable “thyroid pearls”, only five cases exhibited sparse dot-like blood flow signals. This may be attributed to the following factors (1): the “thyroid pearls” were small with limited vascularization, and (2) suboptimal scale adjustment by the operator may have led to inadequate visualization of blood flow.

Herein, most cases (18/20) demonstrated regular morphology, well-defined borders, and no adhesion to surrounding tissues, similar to previous findings (12). Two masses showed adhesion, which may have resulted from compression of adjacent vessels or lymphatics, leading to prolonged tissue fluid exudation. Calcifications were observed in five masses, presenting as punctate or linear hyperechoic echoes, with some displaying comet-tail artifacts. However, since calcifications are commonly seen in mature teratomas, their presence is not specific to the diagnosis of SO.

All patients underwent laparoscopic surgery, which is commonly performed in our hospital due to its minimally invasive advantages. Although some researchers have raised concerns that laparoscopic excision of masses with uncertain pathology may increase the risk of intraoperative spillage and port-site metastasis, advances in laparoscopic techniques have significantly mitigated these risks. All masses were safely enclosed in specimen retrieval bags and completely removed without rupture, consistent with previous research (23, 26). For premenopausal patients who wished to preserve fertility, laparoscopic mass excision was performed, while postmenopausal patients underwent laparoscopic oophorectomy.

The sonographic features of SO, though rare, should be distinguished from other ovarian pathologies. First, unlike ovarian teratomas, which typically present as solid hyperechoic masses with pathognomonic features such as the “doughnut sign” and “fat-fluid level” due to adipose tissue, SO lacks these specific findings. Second, compared to cystadenomas which characterized by thin-walled, unilocular or multilocular cysts with good acoustic transmission, SO often exhibits irregular wall thickening, heterogeneous internal echoes, and mural nodules. Third, endometriomas, typically presenting as thick-walled cystic masses with poor acoustic transmission and uterine adherence, are often accompanied by clinical symptoms such as dysmenorrhea, aiding differentiation. Fourth, Malignant SO is associated with limited diagnostic value on ultrasonography, notably, the presence of a “comet tail” sign should raise suspicion for possible malignant transformation. Finally, simple cysts are typically anechoic with thin, smooth walls, in contrast to the complex internal architecture frequently observed in SO. Familiarity with these distinguishing imaging features, combined with clinical context, is essential for accurate preoperative diagnosis.

Surgery as the mainstay of treatment for SO, the surgical approach should be tailored to each patient (27). In our institution, where all SO were preoperatively diagnosed as benign, the procedure was determined based on fertility preservation needs and patient preference, ten patients underwent total excision of the ovarian mass, while the other ten had mass excision only.

Nonetheless, this study has several limitations. First, it is a retrospective single-center study, and different operators obtained the ultrasound images over an extended period, which may introduce variability and potential bias in image interpretation. Second, due to the rarity of SO, the sample size is small, and the cases span over a long time period. Third, retrospective studies suggest that ultrasound Doppler flow assessment remains inherently subjective, partly due to the absence of robust quantitative parameters. When indicated, contrast-enhanced ultrasound can serve as a valuable adjunct to delineate the extent and distribution of intratumoral neovascularization, thereby improving diagnostic accuracy.

In conclusion, SO is a rare ovarian tumor with nonspecific clinical manifestations. However, ultrasound imaging may reveal characteristic features, typically presenting as unilocular or multilocular cystic or cystic-solid masses with uneven septations and solid protrusions. Some masses may contain hyperechoic solid areas resembling “thyroid pearls,” and Doppler flow signals may be observed within solid components or compartments. Therefore, when adnexal masses exhibit these ultrasound characteristics, SO should be considered in the differential diagnosis.

## Data Availability

The raw data supporting the conclusions of this article will be made available by the authors, without undue reservation.
